# Is social capital protective against hospital readmissions?

**DOI:** 10.1186/s12913-020-05092-x

**Published:** 2020-03-24

**Authors:** Hanna Zlotnick, Geoffrey J. Hoffman, Ushapoorna Nuliyalu, Tedi A. Engler, Kenneth M. Langa, Andrew M. Ryan

**Affiliations:** 1 Princeton University Office of Population Research & Woodrow Wilson School, Princeton, USA; 2 University of Michigan School of Nursing, Ann Arbor, USA; 3 Center for Healthcare Outcomes and Policy, Ann Arbor, USA; 4 Institute for Healthcare Policy and Innovation, Ann Arbor, USA; 5 University of Michigan, School of Public Health, Ann Arbor, USA; 6 Department of Internal Medicine, University of Michigan, Ann Arbor, USA; 7 Veterans Affairs Center for Clinical Management Research, Ann Arbor, USA; 8 Institute for Social Research, Ann Arbor, USA

**Keywords:** Health policy, Cohort analysis, Aging

## Abstract

**Background:**

To evaluate the association between social capital and 30-day readmission to the hospital among Medicare beneficiaries overall, beneficiaries with dementia and related memory disorders, and beneficiaries with dual eligibility for Medicaid.

**Methods:**

Using Health and Retirement Study (HRS) data linked with 2008–2015 Medicare claims from traditional Medicare beneficiaries hospitalized during the study period (1246 unique respondents, 2212 total responses), we examined whether dementia and related memory disorders and dual eligibility were associated with social capital. We then estimated a multiple regression model to test whether social capital was associated with a reduced likelihood of readmission.

**Results:**

Dementia was associated with an − 0.241 standard deviation (sd) change in social capital (95% CI: − 0.378, − 0.103), dual eligibility with a − 0.461 sd change (95% CI: − 0.611, − 0.310), and the occurrence of both was associated with an additional − 0.236 sd change (95% CI: − 0.525, − 0.053). 30-day readmission rates were 14.47% over the study period. In both adjusted and unadjusted models, social capital was associated with small and nonsignificant differences in 30-day readmissions. These effects did not vary across dementia status and socioeconomic status.

**Conclusions:**

Dementia and dual eligibility were associated with lower social capital, but social capital was not associated with the risk of readmission for any population.

## Background

The Hospital Readmission Reduction Program (HRRP) has imposed major financial penalties for hospitals with higher-than-expected readmission rates. Hospitals that care for patients with lower income and education, and higher rates of disability, have been more likely to be penalized, and at greater financial cost, under the program [1, 2]. These penalties have been challenged as unfair, arising from their patients’ greater social and clinical needs that are not captured under standard risk adjustment. In response, Congress modified the penalty formula in the HRRP to take into account differing burdens for hospitals depending on the social complexity of their patient population. Even as the HRRP aims to lessen the burden of penalties borne by hospitals serving a more at-risk population, Congress has not considered the role that factors, like social capital and well-being, play in patients’ recovery and risk of readmission.

Whether certain social capital factors may be protective against readmission, particularly patients with dementia and related memory disorders or of low socioeconomic status, is unknown. Social capital—the synthesis of one’s social integration (or exclusion); social trust; informal and formal social interactions; and engagement in public affairs, volunteerism, and organizational life - may be one such protective factor [3, 4]. Social capital may enhance decision-making, communication, planning, and care networks. All of these factors play significant roles in physician-patient interactions, recovery, and readmission [5–9]. The benefits of social capital may be enhanced for certain patient populations. Patients with dementia have high rates of readmission and frequently suffer from social isolation. Social capital could serve a protective role against readmission for those patients who have strong networks in place [4]. Similarly, among patients of low socioeconomic status, social capital could serve a protective role against readmission because of its capacity to provide meaningful connections and support after discharge that aid in patient-physician communication, the ability to get to appointments, and have a support network to assist with recovery.

This study aims to understand the relationship between social capital factors and hospital readmissions, specifically for patients with dementia and related memory disorders and low socioeconomic status. We use a unique linkage between the Health and Retirement Study (HRS) and Medicare claims to combine detailed survey data on health and social factors with rich clinical data. We hypothesized that social capital would be associated with lower probability of hospital readmission following discharge. We also hypothesized that social capital would lead to greater reductions in readmissions for patients with dementia and lower socioeconomic status.

## Methods

### Data source

We used MedPAR inpatient claims data for Medicare fee-for-service beneficiaries who were discharged between 2008 and 2015. The Medicare Beneficiary Summary File provided beneficiaries’ dual eligible status, and the Chronic Condition Category File provided dementia diagnoses. Beneficiaries’ sociodemographic characteristics, household economic status, and activities of daily living (ADL) difficulties were obtained from the RAND HRS Longitudinal Files and social capital measures were constructed using responses to the biennial HRS Psychosocial & Lifestyle Questionnaires.

The HRS surveys were administered in 2008, 2010, 2012, and 2014. Approximately 80% of Medicare-eligible HRS respondents consent to the linkage of their survey responses to Medicare data. Because the HRS Psychosocial & Lifestyle Questionnaire is only administered to half of the HRS sample every other wave, our sample was reduced substantially. Beneficiary Medicare and HRS data were merged by respondent using the Medicare index discharge date and the nearest HRS survey wave. Hospital readmissions were episode-level, all-cause, 30-day, hospital-wide readmissions.

### Study population

We developed a study cohort that included all U.S. acute care hospital discharges between January 1, 2008 and November 30, 2015 for Medicare beneficiaries who responded to the biennial HRS and HRS Psychosocial & Lifestyle Questionnaire between 2008 and 2014 (1246 unique respondents, 2212 total responses). We excluded discharges from federal hospitals, hospitals in Maryland and Puerto Rico, patients discharged against medical advice, patients who died within thirty days of discharge, and patients who were not continuously enrolled in both Medicare Parts A and Part B between the index admission and 30 days post discharge. We also restricted the cohort to patients who responded to at least 30% of the HRS Psychosocial & Lifestyle Questionnaire questions used to construct our social capital measure.

Our dementia cohort - based on information from the Chronic Condition Warehouse - was defined as patients with a diagnostic history of dementia and related memory disorders prior to their discharge (333 unique respondents, 574 total responses). Our low socioeconomic status cohort was restricted to patients who qualified for both Medicare and Medicaid benefits as assessed using MedPAR dual eligibility indicators (255 unique respondents, 478 total responses). Seventy-six unique respondents (132 total responses) were both dually-eligible and diagnosed with dementia.

### Study design and measures

We used questions in the HRS Psychosocial & Lifestyle Questionnaire to create a composite social capital score based on the Comprehensive Social Capital Index [8]. Responses to these HRS questions served as items for existing scales that indicated six broad social capital factors: social participation and engagement, social network composition, informal sociability, positive social support, social integration, and social cohesion and trust. Because we aimed to measure the protective as opposed to negative potential of social capital, we did not include negative measures of social capital, such as neighborhood disorder or social stressors and demands (Table 1).

**Table 1:** Measures of social capital using the HRS psychosocial and lifestyle questionnaire [9]

Social Capital Component	Target Subject	Items in HRS	Sample Questions
**Social participation & engagement**	**Social Participation - Social Engagement**	**2008: 1a-r**	Please tell us how often you do each activity: Go to a sport, social, or other club?Attend meetings of non-religious organizations, such as political, community, or other interest groups? Work on a hobby or a project? **Score range (for each question):** 1–7 **Maximum raw section score possible:** 126
		**2010–2014: 1a, 1c-i, 1 k-t**	
**Social network composition**	**Composition of Social Network**	**2008–2012: 4, 7, 11, 15**	Do you have a husband, wife, or partner with whom you live? Do you have any living children? Do you have any other immediate family, for example, any brothers or sisters, parents, cousins or grandchildren? Do you have any friends? **Score range (for each question):** 0–1 **Maximum raw section score possible:** 4
		**2014: 3, 6, 10, 14**	
**Informal sociability**	**Contact with Social Network**	**2008–2012: 9a-c, 13a-c, 17a-c**	On average, how often do you do each of the following [refer to contact with children, other family, and friends]: Meet up (include both arranged and chance meetings); speak on the phone; write or email? **Score range (for each question):** 1–6 **Maximum raw section score possible:** 54
		**2014: 8a-c, 12a-c, 16a-c**	
**Positive support**	**Perceived Social Support**	**2008–2012: 5a-c, 8a-c, 12a-c, 16a-c**	**[Refer to relationships with spouse, children, other family, and friends]** How much do they really understand the way you feel about things? How much can you rely on them if you have a serious problem? **Score range (for each question):** 1–4 **Maximum raw section score possible:** 48
		**2014: 4a-c, 7a-c, 11a-c, 15a-c**	
**Social integration**	**Loneliness**	**2008–2012: 20a-k**	How much of the time do you feel: You lack companionship? That there are people you can talk to? That there are people you can turn to? That there are people who really understand you? That there are people you feel close to? Part of a group of friends? **Score range (for each question):** 1–3 **Maximum raw section score possible:** 33
		**2014: 19a-k**	
**Social cohesion & trust**	**Neighborhood Social Cohesion**	**2008–2012: 21a, 21c, 21e, 21 g**	**(These questions ask how you feel about our local area...)** Most people in this area can be trusted/can’t be trusted. If you were in trouble, there are lots of people in this area who would help you/nobody in this area who would help you. **Score range (for each question):** 1–7 **Maximum raw section score possible:** 28
		**2014: 20a, 20c, 20e, 20 g**	

We coded responses and their scales as suggested in the HRS Questionnaire Documentation Report [10]. When appropriate, responses were reverse coded to reflect positive social capital measures (for example, a measure of feeling left out was reverse coded to measure how integrated one felt day-to-day). Questions were categorized according to which of the six broad social capital factors they best represented and responses were aggregated and standardized to measure respondents’ relative personal social capital stock. Individuals received an overall score for each of these six factors, which were then aggregated to create an all-encompassing total social capital score. After the comprehensive social capital score was calculated for each observation, the aggregate total scores were standardized for the analysis. Additional file 1: Table 1 details the six broad social capital factors, contributing HRS questions, and scaling. We evaluated the reliability of the overall social capital scale and the separate subscales using Cronbach’s alpha (Additional file 1: Table 3a & 3b). We found moderate-to-high correlation within as well as across the six categories that comprise the composite social capital score.

Among the observations included in our analysis, levels of missingness in the individual HRS Psychosocial & Lifestyle questions varied from roughly 0 to 15%. Prior to imputation, 55 % of the sample had 100% completion of all social capital indicator questions (*N* = 1212). We imputed missing values for social capital indicators using chained linear multiple imputation methods [2]. We confirmed that imputation yielded generally similar summary statistics to those of non-imputed, fully-complete observations (Additional file 1: Table 4).

### Empirical approach

We first estimated a linear probability model to test the relationship between dementia, dual eligibility, and social capital. The model included indicators for dementia, dual eligibility, and an interaction between these variables. It did not control for other covariates. We estimated this model separately for the summary score of social capital and each subscale. We then estimated a multiple regression linear probability model to test whether social capital was associated with a reduced likelihood of readmission. We estimated separate models for the entire sample, for patients with dementia, and patients with dual-eligibility status to allow the relationships between social capital and readmission to vary across study cohorts. Models accounted for secular time trends and were estimated with and without controls for demographics (age, gender, income, education, Medicaid, race/ethnicity), and relevant health status (body mass index, comorbidities and health history, ADL difficulties). We obtained patient comorbidities and health history from claims data to construct Elixhauser comorbidity summary measures.

## Results

Table 2 shows descriptive statistics for the study sample. Compared to the overall sample, patients with dementia were more likely to be white (85% versus 77%); had somewhat higher difficulties with ADLs (61% versus 45%), and tended to be older (average age of 86 versus 80). Also compared to the overall sample, patients who were dually eligible were more likely to be in the lowest wealth quintile (82% versus 40%); tended to be less educated (have less than a high school degree [53% versus 25%]); and had more functional limitations (had difficulty with one or more ADL) than the overall sample (65% versus 45%).

**Table 2:** Full HRS-CMS readmission model sample characteristics, by cohort 2008–2015: mean (sd)

Variable	All (***N*** = 2212)	Patients with Dementia (***N*** = 574)	Patients who are Dual-Eligible (***N*** = 478)
Years since 2008, years (95% CI)	3.4 (3.3,3.5)	3.4 (3.2,3.6)	3.4 (3.2,3.6)
Readmission N (%)	320 (14.5)	99 (17.2)	76 (15.9)
Unique patients, N	1246	333	255
**Demographics**			
*Self-identified race, % (standard deviation)*			
White	77.1 (42.0)	84.8 (35.9)	46.7 (49.9)
Black/African American	14.0 (34.7)	8.9 (28.5)	27.2 (44.5)
Hispanic	3.6 (18.6)	3.8 (19.2)	10.7 (30.9)
Other	5.3 (22.4)	2.4 (15.4)	15.5 (36.2)
Married/Partnered, % (standard deviation)	40.3 (49.1)	31.4 (46.4)	27.4 (44.7)
Has children, % (standard deviation)	90.7 (29.0)	92.7 (26.1)	88.9 (31.4)
*Wealth quintile, % (standard deviation)*			
Lowest	40.2 (49.0)	43.2 (49.6)	82.0 (38.5)
2nd	27.2 (44.5)	26.1 (44.0)	13.4 (34.1)
3rd	17.5 (38.0)	15.5 (36.2)	3.8 (19.1)
4th	8.6 (28.0)	7.7 (26.6)	0.4 (6.5)
Highest	6.5 (24.7)	7.5 (26.3)	0.4 (6.5)
*Education, % (standard deviation)*			
Less than high school	25.1 (43.4)	26.0 (43.9)	53.3 (49.9)
GED or HS graduate	35.7 (47.9)	37.5 (48.4)	30.3 (46.0)
Some college	22.0 (41.4)	17.9 (38.4)	13.4 (34.1)
College and above	17.1 (37.7)	18.6 (39.0)	2.9 (16.9)
**Disabilities & Care Status**			
*ADL difficulties, % (standard deviation)*			
No difficulties	55.3 (49.7)	39.0 (48.8)	35.4 (47.9)
1–2 difficulties	25.4 (43.5)	29.1 (45.5)	32.4 (46.9)
3+ difficulties	19.3 (39.5)	31.9 (46.6)	32.2 (46.8)
Nursing home resident at time of HRS interview, % (standard deviation)	5.0 (21.8)	14.3 (35.0)	7.1 (25.7)
**Health**			
*BMI, % (standard deviation)*			
Normal	32.1 (46.7)	38.7 (48.7)	30.1 (45.9)
Underweight	3.0 (17.1)	6.1 (23.9)	3.3 (18.0)
Overweight	33.8 (47.3)	35.7 (48.0)	26.6 (44.2)
Obese	31.0 (46.3)	19.5 (39.7)	40.0 (49.0)
*Comorbidities, % (standard deviation)*			
No comorbidities	19.1 (39.3)	16.4 (37.0)	13.0 (33.6)
1–2 comorbidities	54.1 (49.8)	52.8 (50.0)	54.4 (49.9)
3+ comorbidities	26.8 (44.3)	30.8 (46.2)	32.6 (46.9)
Age, years (standard deviation)	79.7 (11.9)	86.0 (8.4)	73.8 (13.9)
Gender, % (standard deviation)	35.3 (47.8)	32.9 (47.0)	24.7 (43.2)

HRS-CMS refers to Health and Retirement Study data linked with administrative claims data from Centers for Medicare and Medicaid Services (CMS). Model includes offset for current CMS readmission risk, which includes age, gender, and medical comorbidities. Model included 29 separate dummy variables for comorbidities, which have been aggregated into categories in this table

**Fig. 1:**
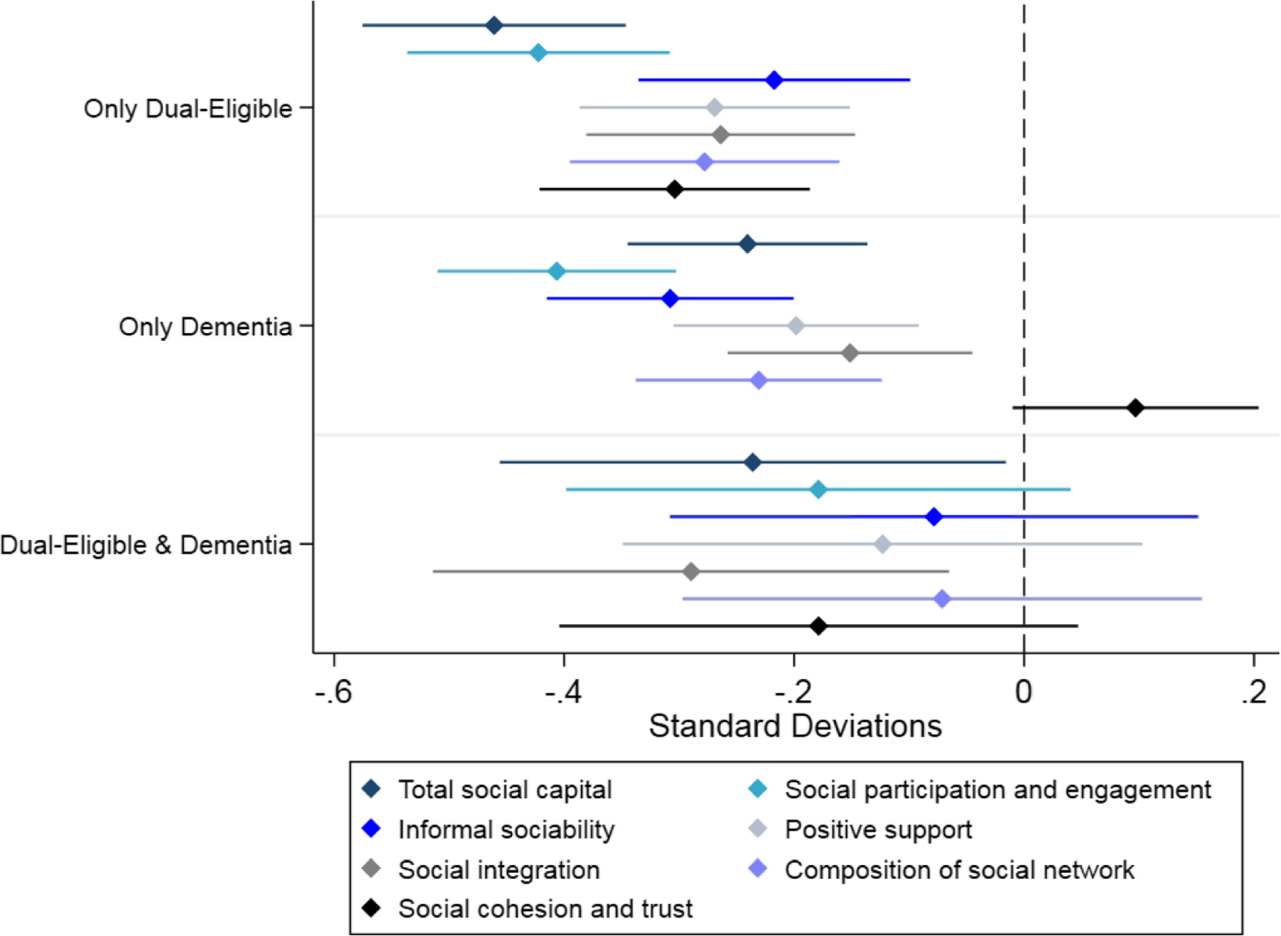
Relationship between dual-eligibility status, dementia, and social risk

Compared to 30-day readmission rates in the overall sample (14.5%), patients with dementia (17.2%) and those who were dually eligible (15.9%) had higher readmission rates (Additional file 1: Table 5). Dementia was associated with a − 0.241 standard deviation (sd) change in social capital (95% CI: − 0.378, − 0.103), dual-eligibility was associated with a − 0.461 sd change in social capital (95% CI: − 0.611, − 0.310), and the joint occurrence of dementia and dual-eligibility was associated with an additional − 0.236 change in social capital (95% CI: − 0.525, − 0.053) (Table 3 and Fig. 1). These results were consistent across all subscales, with the exception of social cohesion where dementia was associated with a non-significant improvement.

**Table 3:** Coefficient estimates showing relationship between dual-eligibility, dementia, and social capital components

Coefficient	Total social capital	Social participation, engagement	Informal sociability	Positive support	Social integration	Social network	Social cohesion, trust
**Dual-Eligible**	−0.461***	− 0.422***	− 0.217***	− 0.269***	− 0.264***	− 0.278***	− 0.304***
	(− 0.611, − 0.310)	(− 0.572, − 0.272)	(− 0.373, − 0.062)	(− 0.424, − 0.114)	(− 0.417, − 0.110)	(− 0.432, − 0.124)	(−0.458, − 0.149)
**Dementia**	− 0.241***	−0.406***	− 0.308***	− 0.198***	− 0.151**	− 0.231***	0.097~
	(− 0.378, − 0.103)	(− 0.543, − 0.270)	(−0.449, − 0.167)	(− 0.338, − 0.058)	(− 0.291, − 0.012)	(− 0.371, − 0.090)	(− 0.044, 0.238)
**Interaction between Dual-Eligible & Dementia**	−0.236*	− 0.179	− 0.078	−0.123	− 0.290*	−0.071	− 0.179
	(−0.525, 0.053)	(− 0.467, 0.110)	(− 0.380, 0.223)	(− 0.420, 0.174)	(− 0.585, 0.005)	(− 0.368, 0.226)	(− 0.475, 0.118)
**Constant**	0.184***	0.208***	0.140***	0.127***	0.116***	0.134***	0.052~
	(0.114, 0.253)	(0.139, 0.277)	(0.069, 0.212)	(0.055, 0.199)	(0.045, 0.186)	(0.063, 0.205)	(− 0.019, 0.123)

95% confidence intervals in parentheses. *** *p* < 0.001, ** *p* < 0.01, * *p* < 0.05, ~ *p* < 0.10. Results are from a regression with two dummies (dual-eligible and dementia) and an interaction between them. Models include 2212 observations

Table 4 shows the relationship between the model variables and 30-day readmissions. In unadjusted models, a one-sd increase in social capital was associated with a nonsignificant − 0.004 difference in 30-day readmission (95% CI: (− 0.019, 0.011)). In adjusted models, a one-sd increase in social capital was associated with a nonsignificant 0.002 difference in 30-day readmission (95% CI: (− 0.020, 0.024)). The association between social capital and readmissions did not vary across dementia and socioeconomic status: among patients with dementia, a one-sd increase in social capital was associated with a nonsignificant 0.027 difference in 30-day readmission (95% CI:(− 0.020, 0.074)); among patients with dual eligibility, a one-sd increase in social capital was associated with a nonsignificant 0.007 difference in 30-day readmission (95% CI:(− 0.052, 0.037)).

**Table 4:** Relationship between model variables and 30-day readmission rates

Coefficient	All (***N*** = 2212)	Patients with Dementia (***N*** = 574)	Patients who are Dual-Eligible (***N*** = 478)
Years since 2008	0.001 (− 0.008,0.011)	− 0.000 (− 0.021,0.021)	0.001 (− 0.021,0.024)
Black/African American	0.007 (− 0.054,0.069)	0.154* (− 0.001,0.309)	− 0.037 (− 0.157,0.084)
Hispanic	0.056 (− 0.055,0.168)	0.183* (− 0.048,0.414)	−0.037 (− 0.211,0.137)
Other	− 0.024 (− 0.117,0.068)	−0.022 (− 0.297,0.254)	−0.056 (− 0.201,0.089)
Married/Partnered?	− 0.040* (− 0.091,0.011)	−0.011 (− 0.122,0.101)	−0.013 (− 0.149,0.123)
Has children?	0.040 (− 0.028,0.109)	0.028 (− 0.136,0.193)	0.035 (− 0.111,0.182)
**Income quintile**			
2nd	0.023 (−0.029,0.076)	0.066 (− 0.048,0.180)	−0.001 (− 0.153,0.151)
3rd	0.013 (− 0.051,0.077)	−0.057 (− 0.194,0.080)	0.166~ (− 0.088,0.421)
4th	−0.010 (− 0.091,0.071)	−0.034 (− 0.219,0.150)	−0.074 (− 0.758,0.610)
5th (highest)	0.041 (− 0.054,0.137)	0.023 (− 0.172,0.218)	−0.174 (− 0.870,0.522)
**Education**			
GED or HS graduate	−0.025 (− 0.079,0.029)	−0.046 (− 0.160,0.069)	−0.075~ (− 0.191,0.041)
Some college	− 0.031 (− 0.092,0.030)	−0.017 (− 0.155,0.121)	−0.117* (− 0.268,0.034)
College and above	− 0.034 (− 0.105,0.036)	−0.014 (− 0.165,0.138)	−0.113 (− 0.386,0.160)
Total social capital	0.002 (− 0.020,0.024)	0.027 (− 0.020,0.074)	−0.007 (− 0.052,0.037)
Constant	− 0.025 (− 0.222,0.172)	0.028 (− 0.497,0.552)	0.085 (− 0.326,0.496)

Confidence intervals in parentheses. * *p* < 0.05, ~ *p* < 0.10. Model included health status (BMI, ADLs, comorbidities), age, and gender. Income quintiles vary by year and were included in the model but excluded from t created using Household Income Quintiles data from Urban Institute & Brooking Institution Tax Policy Center [10]. Upper limits for quintiles are as follows: 2008: Q1: $20.712, Q2: $39,000, Q3: $62,725, Q4: $100,240, Q5: >$100,240. 2010: Q1: $20,000, Q2: $38,000, Q3: $61,500, Q4: $100,029, Q5: >$100,029. 2012: Q1: $20,599, Q2: $39,764, Q3: $64,582, Q4: $104,096, Q5: >$104,096. 2014: Q1: $21,432, Q2: $41,186, Q3: $68,212, Q4: $112,262, Q5: >$112,262

## Discussion

In our national study of the relationship between social capital and readmissions among Medicare patients, we identified three main findings. First, patients who had dementia and who were also eligible for Medicaid had lower levels of social capital. Second, as in other studies about social risk and readmission, we found that patients with lower educational attainment and lower income had higher risk of readmissions. Third, in both adjusted and unadjusted models, we found that social capital was not associated with readmission rates. The relationship between social capital and readmission also did not vary meaningfully across patients with dementia and those with dual eligibility for Medicare and Medicaid.

Our findings extend other research evaluating the association between social risk and readmission rates. One prior using the same data sources found that including social factors marginally improved risk prediction for 30-day readmission [8]. Our study extended this work by examining the relationship between social capital and readmission, which has not been previously explored in the literature. Other research shows that tangible social risk, such as ADL limitations reflecting disability and limited social roles, is an important contributor to readmissions that is not captured in existing Medicare risk-adjustment [1]. Moreover, observed relationships between socioeconomic status, wealth, and readmission suggest that certain patients might have cumulative social risk that interferes with optimal post-discharge self-management [6, 11]. In contrast, our study and its findings on social capital across socioeconomic status and vulnerable patient types, suggests that capital itself is not the explanation for a post-discharge advantage in self-management. Rather, prior findings on readmission and social risk, including socioeconomic status, may reflect area factors (such as neighborhood cohesion and opportunity for engagement) and personal characteristics (strong support networks, activity in the community, sense of belonging) that may be responsible for these disparities in post-discharge self-management and consequential readmission risk.

Previous research by Herrin et al. [12] on community factors suggested other explanations for observed links between readmission and social factors such as socioeconomic status. They identified strong associations between higher readmission rates and access to care, which is strongly associated with a county’s socioeconomic status, just as social capital and community opportunity are tightly tied to the community’s socioeconomic status. Further, when Calvillo-King et al. [3] explored the impact of social factors on readmissions, results showed that post-discharge readmission was affected by nuanced elements of the social environment, individual behavioral and socio-cognitive factors, and neighborhood factors.

Along with evidence from prior research, our findings suggest that functional disability and access to timely, high-quality primary care are relatively more important determinants of readmissions than accumulated social risk. While social capital is relevant to many health outcomes, hospital readmission may be more proximally related to other clinical and structural factors. Quality of life and well-being outcomes may be more likely to be related to social capital because of the stronger direct impact of social support and community integration on care networks and quality of life for long-term diagnoses and health challenges.

Our study has a number of important limitations. First, up to 15% of responses to survey items from the HRS Psychosocial & Lifestyle Questionnaire were missing. After imputing these missing values, we found strong alignment between imputed results with non-imputed data to ensure that results aligned. Nonetheless, if social capital is most protective for those in poor health, who are more likely to drop out of the HRS, we would be more likely to see these null results. Second, our results may be confounded by unobserved factors that we did not include in the model. For example, regardless of one’s social capital at any level of income, there may be additional/influential social characteristics and constraints that inhibit these patients from optimizing self-care upon discharge, such as lack of self-mastery. Self-mastery should be positively associated with social capital, and negatively associated with readmissions [11, 13]. By not controlling for it, we may be pushing the observed association away from the null and toward a protective relationship. However, our null results suggest that this concern is not likely to be a major source of bias. Finally, our results may not generalize to non-elderly patients who are not enrolled in the Medicare program.

## Conclusion

Despite the null findings, our results have policy implications for evaluating less tangible factors such as social capital that may influence health outcomes. First, our study suggests that the recent CMS change to the HRRP policy, which addresses hospitals’ populations of dual eligible patients, where dual eligibility is strongly correlated with disability, may be the proper approach to address hospital-level disparities in readmission penalties. Second, regardless of how CMS’s restructuring of penalties on care for dually eligible patients plays out, clarifying the pathways linking socioeconomic status and readmissions remains an important challenge. Dissecting this relationship and analyzing how social capital, social networks, and community characteristics affect health outcomes are crucial to designing well-informed interventions to improve care. This analysis has also shown that patients with dementia and low socioeconomic status have low capital, putting them at risk for other adverse health events. Future research on how social capital influences longer term health outcomes and measures of well-being and quality of life may provide a more vivid picture of how the synthesis of one’s social engagement, trust, networks, and support could be targeted at community levels to enhance self-care, physician-patient relations, and trust and following of preventive measures and health advice.

## Supplementary information

**Additional file 1: Table 1**. Measures of Social Capital Using the HRS Psychosocial and Lifestyle Questionnaire (In-Depth) [9]. **Table 2**. Missingness of social capital indicator HRS questions pre-imputation, all years. **Table 3**a. Cronbach Alpha (HRS Psychosocial & Lifestyle Questionnaire Questions). **Table 3**b. Cronbach Alpha (Social Capital Composite Score Categories). **Table 4**. HRS Questionnaire sample characteristics pre- and post-imputation 7. **Table 5**. Sample Characteristics - Social capital components and readmissions 2008–2015. **Figure 1**a. Distribution of Social Capital – All Patients. **Figure 1**b. Distribution of Social Capital – Patients with Dementia. **Figure 1**c. Distribution of Social Capital – Dually-Eligible Patients.

## Data Availability

The datasets used and/or analysed in this study are protected health information and not available to share.

## Abbreviations

ADL: Activities of daily living
CI: Confidence interval
CMS: Centers for Medicare & Medicaid Services
HRRP: Hospital Readmission Reduction Program
HRS: Health and Retirement Study
MedPAR: Medicare Provider Analysis and Review
sd: Standard deviation
