# Supplementary material for: Is social capital protective against hospital readmissions?
Source: BMC Health Serv Res. 2020 Mar 24;20:248. doi: 10.1186/s12913-020-05092-x (PMC7092426; doi:10.1186/s12913-020-05092-x)

**Supplemental Appendix**

**Contents**

[**Table 1. Measures of Social Capital Using the HRS Psychosocial and Lifestyle Questionnaire (In-Depth) (9)** 3](#_Toc26484)

[**Table 2. Missingness of social capital indicator HRS questions pre-imputation, all years** 4](#_Toc26485)

[**Table 3a. Cronbach Alpha (HRS Psychosocial & Lifestyle Questionnaire Questions)** 5](#_Toc26486)

[**Table 3b. Cronbach Alpha (Social Capital Composite Score Categories)** 6](#_Toc26487)

[**Table 4. HRS Questionnaire sample characteristics pre- and post-imputation** 7](#_Toc26488)

[**Table 5. Sample Characteristics - Social capital components and readmissions 2008-2015** 8](#_Toc26489)

[**Figure 1a. Distribution of Social Capital – All Patients** 9](#_Toc26490)

[**Figure 1b. Distribution of Social Capital – Patients with Dementia** 9](#_Toc26491)

[**Figure 1c. Distribution of Social Capital – Dually-Eligible Patients** 9](#_Toc26492)

### **Table 1. Measures of Social Capital Using the HRS Psychosocial and Lifestyle Questionnaire (In-Depth) (9)**

| **Social Capital Component** | **Target Subject** | **Contributing Questions** | **Questions** |
| --- | --- | --- | --- |
| Social participation & engagement | Social Participation - Social Engagement | *2008:* 1a-r  *2010-2014:*  1a, 1c-i, 1k-t | *Please tell us how often you do each activity: Care for a sick or disabled adult? Do volunteer work with children or young people? Do any other volunteer or charity work? Attend an educational or training course? Go to a sport, social, or other club? Attend meetings of non-religious organizations, such as political, community, or other interest groups? Pray privately in places other than a church or synagogue? Read books, magazines, or newspapers? Do word games such as crossword puzzles or Scrabble? Play cards or games such as chess? Do writing (such as letters, stories, or journal entries? Use a computer for email, Internet or other tasks? Do home or car maintenance or gardening? Bake or cook something special? Make clothes, knit, embroider, etc.? Work on a hobby or a project? Play sports or exercise? Walk for 20 minutes or more?*  ***Score range (for each question):*** 1-7 **Maximum raw section score possible:** 126 |
| Social network composition | Composition of Social Network | *2008-2012:*  4, 7, 11, 15  *2014:* 3, 6, 10, 14 | *Do you have a husband, wife, or partner with whom you live? Do you have any living children? Do you have any other immediate family, for example, any brothers or sisters, parents, cousins or grandchildren? Do you have any friends?*  ***Score range (for each question):*** 0-1 **Maximum raw section score possible:** 4 |
| Informal sociability | Contact with Social Network | *2008-2012*: 9a-c, 13a-c, 17a-c  *2014:* 8a-c, 12a-c, 16a-c | *On average, how often do you do each of the following [refer to contact with children, other family, and friends]: Meet up (include both arranged and chance meetings); speak on the phone; write or email?*  ***Score range (for each question):*** 1-6 **Maximum raw section score possible:** 54 |
| Positive support | Perceived Social Support | *2008-2012:* 5a-c, 8a-c, 12a-c, 16a-c  *2014:* 4a-c, 7a-c, 11a-c, 15a-c | *[Refer to relationships with spouse, children, other family, and friends] How much do they really understand the way you feel about things? How much can you rely on them if you have a serious problem? How much can you open up to them if you need to talk about your worries?*  ***Score range (for each question):*** 1-4 **Maximum raw section score possible:** 48 |
| Social integration | Loneliness | *2008-2012:* 20a-k  *2014:* 19a-k | *How much of the time do you feel: You lack companionship? Left out? Isolated from others? That you are “in tune” with the people around you? Alone? That there are people you can talk to? That there are people you can turn to? That there are people who really understand you? That there are people you feel close to? Part of a group of friends? That you have a lot in common with the people around you?*  ***Score range (for each question):*** 1-3 **Maximum raw section score possible:** 33 |
| Social cohesion & trust | Neighborhood Social Cohesion | *2008-2012:* 21a, 21c, 21e, 21g  *2014:*  20a, 20c, 20e, 20g | *(These questions ask how you feel about our local area: that is everywhere within a 20 minute walk or about a mile from your home) I really feel a part of this area/I feel that I don’t belong in this area. Most people in this area can be trusted/Most people in this area can’t be trusted. Most people in this area are friendly/Most people in this area are unfriendly. If you were in trouble, there are lots of people in this area who would help you/If you were in trouble, there is nobody in this area who would help you.*  ***Score range (for each question):*** 1-7 **Maximum raw section score possible:** 28 |

### **Table 2. Missingness of social capital indicator HRS questions pre-imputation, all years**


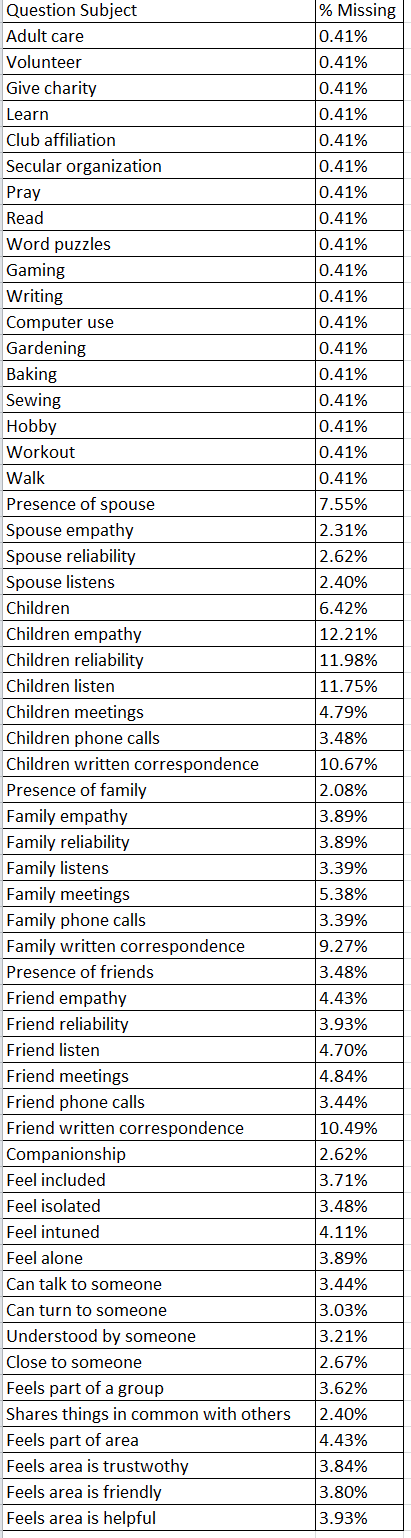


### **Table 3a. Cronbach Alpha (HRS Psychosocial & Lifestyle Questionnaire Questions)**


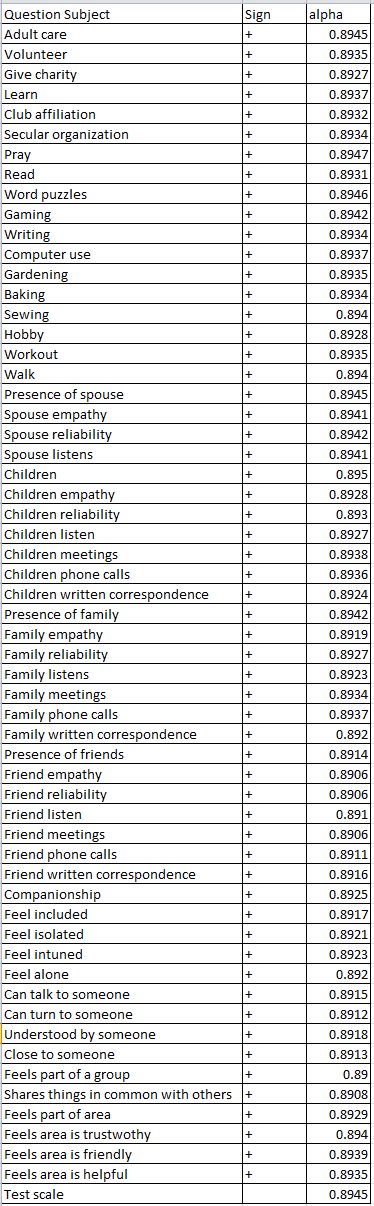


### **Table 3b. Cronbach Alpha (Social Capital Composite Score Categories)**


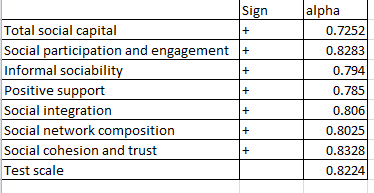


### **Table 4. HRS Questionnaire sample characteristics pre- and post-imputation**


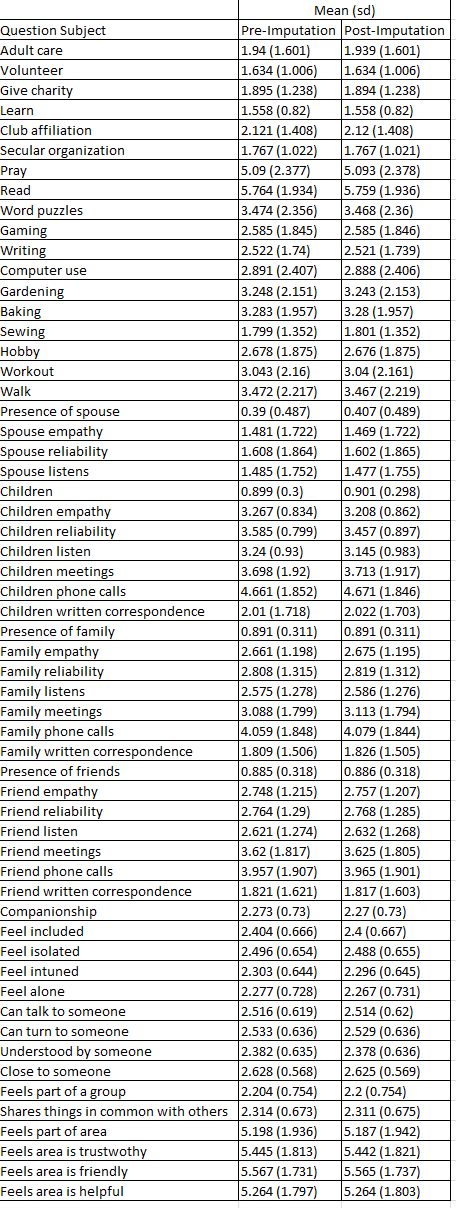


### **Table 5. Sample Characteristics - Social capital components and readmissions 2008-2015**

|  | All (N=2212) | Dementia (N=574) | Dual-Eligible (N=478) |
| --- | --- | --- | --- |
| Rate of readmission, % (standard deviation) | 14.5 (35.2) | 17.2 (37.8) | 15.9 (36.6) |
| Total social capital, mean (standard deviation) | 0.008 (0.995) | -0.217 (1.076) | -0.409 (1.045) |
| Social participation and engagement | 0.001 (0.999) | -0.337 (1.000) | -0.376 (1.001) |
| Informal sociability | 0.009 (0.998) | -0.235 (1.055) | -0.184 (1.082) |
| Positive support | 0.010 (0.993) | -0.162 (1.045) | -0.231 (1.050) |
| Social integration | 0.002 (0.999) | -0.163 (1.104) | -0.270 (1.086) |
| Social network | 0.010 (0.993) | -0.177 (1.060) | -0.227 (1.011) |
| Social cohesion and trust | 0.001 (1.000) | 0.038 (1.070) | -0.274 (1.068) |

***Note:*** *Total social capital score and component scores standardized to mean = 0, standard deviation = 1.*

### **Figure 1a. Distribution of Social Capital – All Patients**


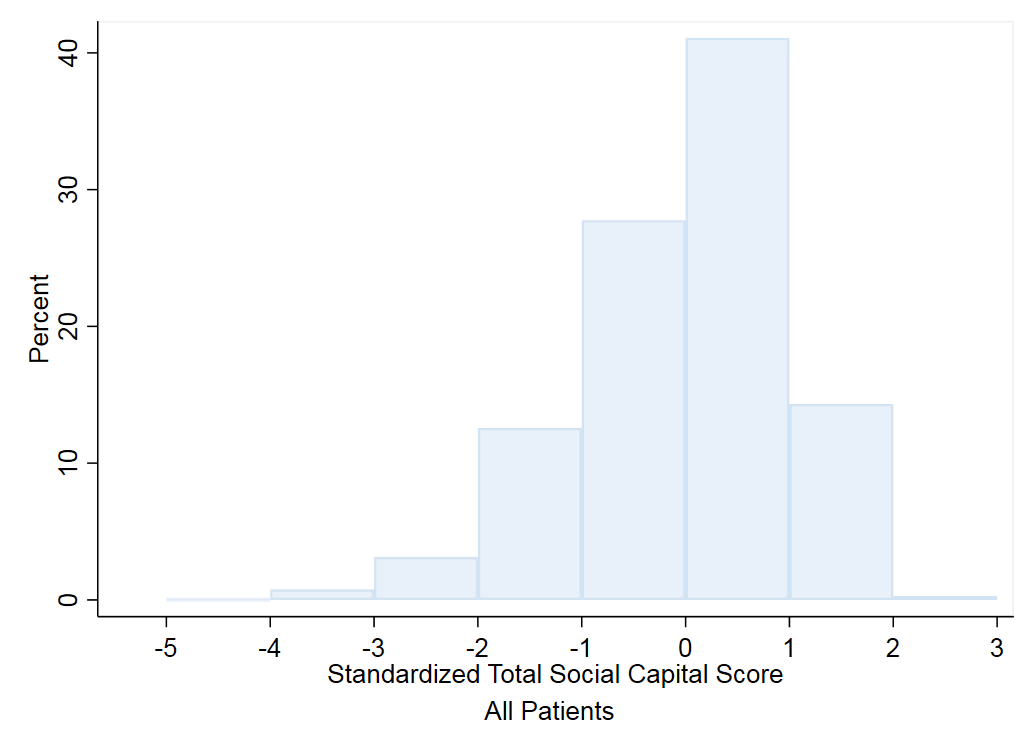


### **Figure 1b. Distribution of Social Capital – Patients with Dementia**


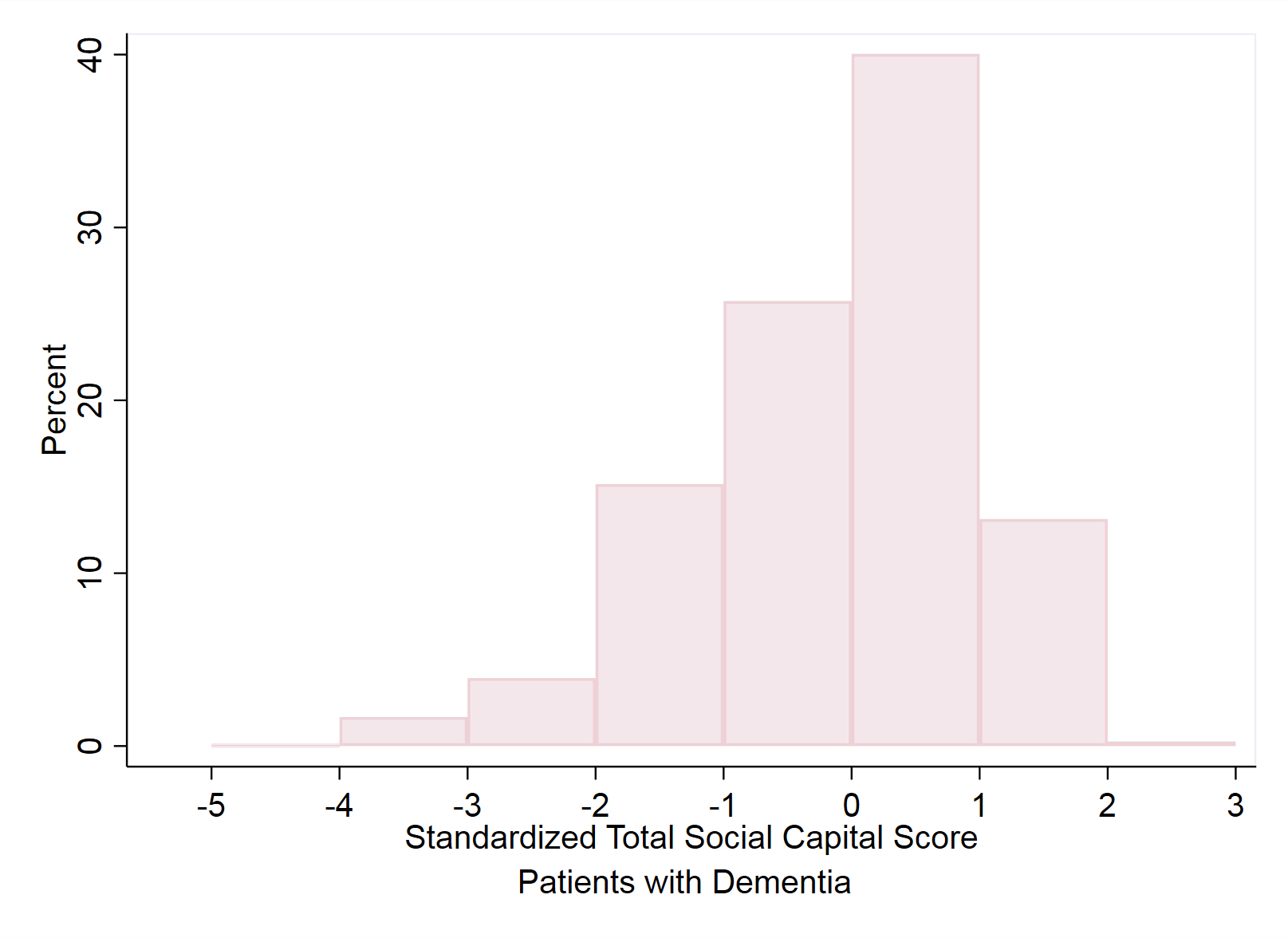


### **Figure 1c. Distribution of Social Capital – Dually-Eligible Patients**


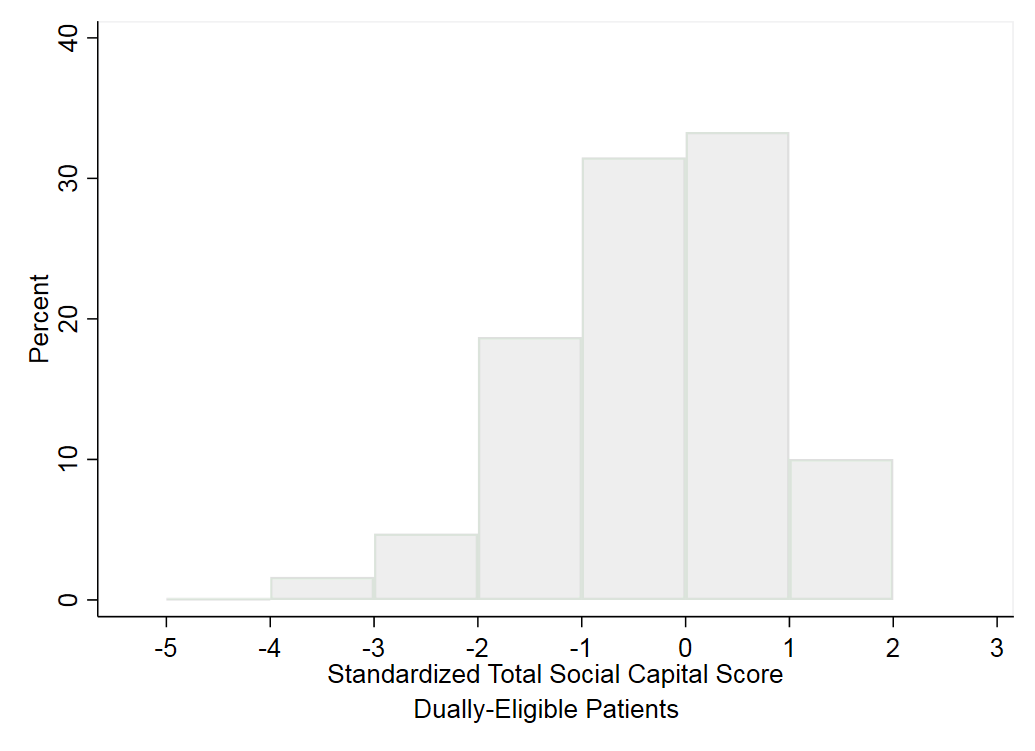

Supplement: Supplementary file 1 — Additional file 1: Table 1. Measures of Social Capital Using the HRS Psychosocial and Lifestyle Questionnaire (In-Depth) [9]. Table 2. Missingness of social capital indicator HRS questions pre-imputation, all years. Table 3a. Cronbach Alpha (HRS Psychosocial & Lifestyle Questionnaire Questions). Table 3b. Cronbach Alpha (Social Capital Composite Score Categories). Table 4. HRS Questionnaire sample characteristics pre- and post-imputation 7. Table 5. Sample Characteristics - Social capital components and readmissions 2008–2015. Figure 1a. Distribution of Social Capital – All Patients. Figure 1b. Distribution of Social Capital – Patients with Dementia. Figure 1c. Distribution of Social Capital – Dually-Eligible Patients. [file 12913_2020_5092_MOESM1_ESM.docx]
